# Investigations on Adhesion Characteristics between High-Content Rubberized Asphalt and Aggregates

**DOI:** 10.3390/polym14245474

**Published:** 2022-12-14

**Authors:** Xiaofeng Wang, Jianan Liu, Zhenjun Wang, Haosen Jing, Bo Yang

**Affiliations:** 1 Henan Provincial Communications Planning & Design Institute, Zhengzhou 450052, China; 2 School of Materials Science and Engineering, Chang’an University, Xi’an 710061, China; 3 Engineering Research Center of Pavement Materials, Ministry of Education of China, Chang’an University, Xi’an 710064, China

**Keywords:** rubberized asphalt, adhesion characteristics, binder bond strength (BBS), surface free energy (SFE), atomic force microscope (AFM)

## Abstract

The use of waste tires to prepare rubberized asphalt has been a hot trend in recent years, and the characteristics of adhesion between rubberized asphalt and aggregates are important factors affecting the performance of asphalt pavement. However, there is a lack of uniform results on the adhesion characteristics of rubberized asphalt. Therefore, crumb-rubber-modified asphalt (CRMA) with 15%, 20%, and 25% rubber contents was prepared in this work, and the basic rheological parameters and cohesive energy of the rubberized asphalt were characterized by DSR. The adhesion properties between rubberized asphalt and aggregates were characterized based on macroscopic binder bond strength (BBS), surface free energy (SFE) theory, and nanoscale atomic force microscopy (AFM) tests. The results show that crumb rubber (CR) can improve the high-temperature elastic properties of asphalt; secondly, CR can have a negative impact on the maximum tensile strength of asphalt and aggregates. CR can improve the SFE parameter of asphalt. The work of adhesion of rubberized asphalt and limestone is the highest, followed by basalt and, finally, granite. Finally, CR can cause the catanaphase in asphalt to gradually break down and become smaller, and the adhesion of rubberized asphalt can be reduced. Overall, CR can reduce the adhesion performance of asphalt, and this work provides a reference for the application of rubberized asphalt.

## 1. Introduction

With the continuous increase in car ownership, the disposal of waste tires has become a pressing issue for scholars from all over the world [1]. The crumb rubber (CR) prepared by recycling and reprocessing waste tires can be used as a modifier in asphalt binders [2,3]. CR powder can not only improve various properties of asphalt binders, but also significantly improve the environmental problems caused by waste tires [3]. As a typical solid waste product, CR powder improves the sustainability of road development. CR powder has great potential in the research of road materials and related fields because of its huge output, excellent performance, and environmental protection advantages [4,5,6,7].

The properties of CR-powder-modified asphalt are affected by many factors, including the particle size of the CR powder, the type of CR powder molding, the CR content, the type of blending, the pretreatment process, etc. Xiao Feipeng et al. focused on the plasma treatment of CR powder, and the internal de-crosslinking process of the CR powder improved the compatibility between the CR powder and asphalt [8,9]. The compatibility of CR powder and asphalt can also be enhanced by using CR powder desulfurized by microwaves, or by adding waste oil containing more light components in the process of CR powder modification of asphalt, and the rheological properties of crumb-rubber-modified asphalt (CRMA) can be improved [10,11]. The particle size of CR powder can affect the rheological properties of CRMA, and larger CR powder particles can help to enhance the fatigue performance of CRMA [12]. Some scholars have used graphene/carbon black composite materials and CR powder to create composite-modified asphalt, and the results show that the rutting resistance and healing properties of this composite-modified asphalt were improved [13]. In addition, the blending compatibility of CRMA and waste plastics and the aging resistance of CRMA have also been research hotspots in recent years [14,15].

Asphalt pavement is repeatedly affected by traffic loads and the environment during its use. Especially in a moisture-immersed state, the adhesion between the asphalt and aggregate may fail. The aggregate can fall off and the performance of the asphalt pavement will be seriously deteriorated [16,17,18]. This situation not only increases the cost of road maintenance, but also causes security risks [19]. However, the results of research on the moisture damage resistance of CRMA are inconsistent. Through static contact angle studies, Zahid Hossain et al. found that the incorporation of CR powder can improve the surface energy of the binder, improve the viscosity of the binder, and reduce the penetration value of the binder, showing a positive effect on resistance to moisture damage [20]. M.N. Partl et al. used CR powder to prepare an open-graded asphalt mixture and conducted a coaxial shear test (CAST), and the study found that compared with traditional porous or semi-porous asphalt mixtures, the moisture sensitivity of the CRMA mixture was reduced [21]. However, there are also different viewpoints. Quan Lv et al. studied the pull-off tensile strength of CRMA, polymer-modified asphalt, and matrix asphalt and basalt slabs on a large scale through binder bond strength (BBS) tests, on the basis of controlling the asphalt film thickness [22,23,24,25]. It was found that the polymer and CR powder adversely affected the pull-off tensile strength of the asphalt aggregates.

In addition to the common water boiling test, water immersion test, surface free energy theory, and BBS test, atomic force microscopy (AFM) for nanoscale research has also developed rapidly in recent years. The microstructural characteristics of asphalt surfaces show multinomial heterogeneity at the nanoscale, and the nanoscale properties of asphalt have always been the focus of academic research [26,27,28]. AFM is a powerful tool for evaluating the microstructure of asphalt. In 1996, L. Loeber et al. first used AFM to discover the bee structure of the asphalt surface [29]. The bee structure is also called catanaphase in the later research classification, which is temperature-reversible [28]. The chemical composition of the catanaphase was initially thought to be mainly asphaltenes, but increasing evidence suggests that interactions between wax crystals and other chemical constituents in asphalt lead to the formation of the catanaphase [30]. AFM is often used to evaluate asphalt’s modification effects, degree of aging, and adhesion [31,32,33].

The loss of adhesive bonds and the fracture of cohesive bonds under the action of water are the main causes of moisture damage [34]. Common adhesion theories include chemical reaction, surface energy, molecular orientation, and mechanical adhesion [35]. When the deformation exceeds the influence of mechanical interlocking and surface molecular orientation, cohesive bond failure occurs [36]. Conventional adhesive strength tests such as the pneumatic adhesion tensile testing instrument (PATTI) and BBS have certain limitations and have been continuously improved [23]. The compression pull-off test has been developed and proven to be excellent [37]. Considering the complexity of adhesion and the diversity of test methods, the adhesion mechanism of crumb-rubber-modified asphalt needs to be further explored.

In this work, the rheological parameters of asphalt binder and base asphalt were characterized for three CR powder contents. The pull-off tensile strength between asphalt with di and limestone, basalt, and granite slabs was tested, and the effect of crumb rubber powder on the BBS was analyzed. Secondly, the SFE parameters of asphalt binders with different crumb rubber powder contents were studied, and the work of adhesion between different asphalt binders and three aggregates was calculated. In addition, the changes in the catanaphase and the adhesion force were used to analyze the influence of the modification of the CR powder on the adhesion and its mechanism of action via AFM. Finally, the effects of CR powder on the adhesion between the binders and aggregates were compared and analyzed from the perspectives of macroscopic strength, surface free energy theory, and nanomechanical properties. The results of this research can help to understand the effect of CR powder on the moisture damage resistance of the mixture, so as to ensure the long-term durable use of the pavement.

## 2. Materials and Methods

### 2.1. Materials

The base asphalt was used in this work, and its properties were tested according to the “Standard Test Methods of Asphalt and Bituminous Mixtures for Highway Engineering” (JTG E20-2011) [38], as shown in Table 1. The CR powder used in this work was 80 mesh, its relative density was 1.128, its water content was 0.49%, and its metal content and sieve residue were 0.02% and 4.27%, respectively. Distilled water, ethylene glycol, and glycerol were used to measure the contact angles of asphalt with different rubber powder contents and aggregates, and the surface energies of the three liquids are shown in Table 2 [39]. The detailed parameters can be found in Section 2.3.3. The chemical composition of the aggregates is shown in Table 3.

### 2.2. Preparation of Rubberized Asphalt Binder

The asphalt was heated and melted, and then CR powder with a mass of 15%, 20%, or 25% of the asphalt was added. The asphalt was then sheared at 4000 rpm for 60 min at 180 °C, followed by low-speed stirring for 30 min (800 rpm) [23]. The base asphalt was named 90#, and the three rubberized asphalts were named CR-15, CR-20, and CR-25.

### 2.3. Methodology

#### 2.3.1. Dynamic Shear Rheometer (DSR) Test

The Anton Paar SmartPave 102 DSR was used to test the rheological parameters of the different asphalts. The test adopted a temperature sweep; the temperature range was from 46 °C to 82 °C. The test was performed once at an interval of 6 °C; the frequency was 10 rad/s, and the strain was controlled to 1.5% to ensure that the asphalt’s rheological behavior was within the linear viscoelastic (LVE) range [40,41]. Three replicate experiments were performed on the same sample to eliminate accidental errors.

#### 2.3.2. Binder Bond Strength (BBS) Test

**Figure 1:**
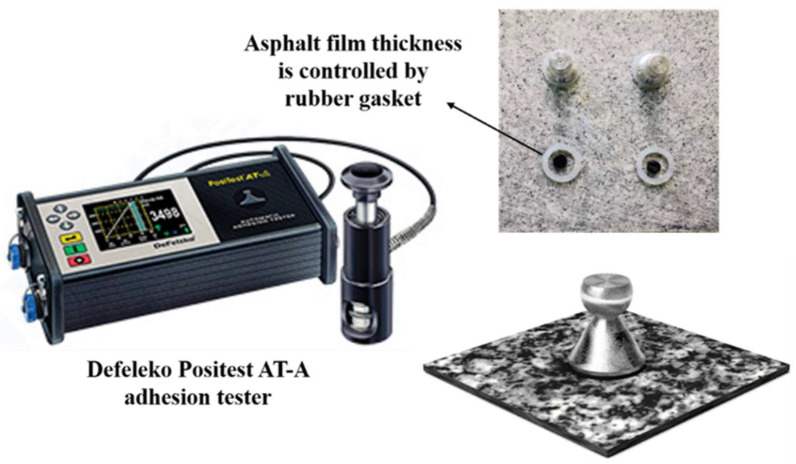
The process of the BBS test.

The BBS test is conducted based on AASHTO TP-91 [42], and a American Defelsko Positest AT-A adhesion tester was used to evaluate the binder bond strength of different kinds of asphalt. The diameter of the stub was 20 mm and the tensile strength loading rate was 0.7 MPa/s. The thickness of the asphalt film was maintained by the crumb rubber gasket at 0.8 mm [23]. At this time, in addition to adhesion failure, cohesive ductile damage still interfered. The aggregates’ base materials were limestone, basalt, or granite, as shown in Figure 1. Prior to the test, each specimen was subjected to 48 h of moisture conditioning in a 40 °C water bath. In water conditioning, 15 h of conditioning can affect the bond strength and failure mode. As a result, most samples exhibited an adhesive failure. The peak tensile strength was recorded to quantitatively evaluate the adhesion properties between the different asphalts and aggregates [25]. Pull-off tensile strength (POTS) is the maximum tensile strength of the stub pulling away from the aggregates in the BBS test.

#### 2.3.3. Surface Free Energy (SFE) Theory

The surface energy consists of two parts: the dispersion component and the polar component. The expression is shown in Equation (1).

(1) $$\gamma =\gamma^{d}+\gamma^{p}$$

where γ is the surface free energy (mJ/m^2^), $\gamma^{d}$ is the dispersive component (mJ/m^2^), and $\gamma^{p}$ is the polarity component (mJ/m^2^).

(2) $$W_{a}=2\gamma$$

The cohesion energy or cohesive bond energy ($W_{a}$) is defined as the value of the energy needed to create two new surfaces with unit areas [43,44]. A higher value of cohesion energy implies a higher level of energy needed for propagating a crack and fracturing the material into two new surfaces [45].

The surface energy of asphalt can be calculated using Equations (3)–(5), which can be obtained from Young’s equation and surface energy theory [46].

(3) $$\gamma_{l}\cos\theta =\gamma_{s}-\gamma_{sl}$$

(4) $$\gamma_{sl}=\gamma_{s}+\gamma_{l}-2\sqrt{\gamma_{s}{}^{d}\gamma_{l}{}^{d}}-2\sqrt{\gamma_{s}{}^{p}\gamma_{l}{}^{p}}$$

(5) $$\frac{1+\cos\theta }{2}\frac{\gamma_{l}}{\sqrt{\gamma_{l}{}^{d}}}=\sqrt{\gamma_{s}{}^{p}}\sqrt{\frac{\gamma_{l}{}^{p}}{\gamma_{l}{}^{d}}}+\sqrt{\gamma_{s}{}^{d}}$$

where $\gamma_{s}$, $\gamma_{l}$, and $\gamma_{sl}$ are the surface free energy of the solid, liquid, and solid–liquid phases, respectively (mJ/m^2^); $\gamma_{l}{}^{d}$ and $\gamma_{l}{}^{p}$ express the dispersion component and polar component of the surface energy of the liquid phase, respectively (mJ/m^2^); $\gamma_{s}{}^{p}$ and $\gamma_{s}{}^{d}$ are the dispersion component and polar component of the solid (asphalt) phase, respectively (mJ/m^2^); θ is the angle connecting the solid–liquid interface; and $\gamma_{b}$ is the surface free energy of the aggregate (mJ/m^2^).

In Equation (5), $\frac{(1+\cos\theta )\gamma_{l}}{2\sqrt{\gamma_{l}{}^{d}}}$ can be regarded as the *y* coordinate and $\sqrt{\frac{\gamma_{l}{}^{p}}{\gamma_{l}{}^{d}}}$ can be regarded as the *x* coordinate. The measured contact angle and the surface energy data of the three liquids can be substituted into the *x* and *y* coordinates for linear fitting. The square of the slope of the fitting line is the polar component of the solid surface energy. The square of the fitting intercept is the dispersion component of the surface energy. The sum of the two is the total surface energy.

The work of adhesion is used to evaluate the difficulty of water penetrating the asphalt film into the binder–aggregate interface of the asphalt mixture, and the work of adhesion for asphalt–aggregate systems can be calculated as shown in Equation (6):

(6) $$W_{as}=\gamma_{l}(1+\cos\theta )$$

where $W_{as}$ is the work of adhesion between the asphalt and the solid (limestone) (mJ/m^2^).

In the actual measurement, the actual heating temperature, drop height, and droplet size of the asphalt are difficult to control, so Equation (7) can be used to calculate the asphalt–aggregate adhesion work.

(7) $$W_{as}=2\left(\sqrt{\gamma_{s}{}^{d}\gamma_{a}{}^{d}}+\sqrt{\gamma_{s}{}^{p}\gamma_{a}{}^{p}}\right)$$

where $\gamma_{s}{}^{d}$ and $\gamma_{s}{}^{p}$ express the dispersion component and polar component of the surface energy of the solid (limestone) phase, respectively (mJ/m^2^), while $\gamma_{a}{}^{p}$ and $\gamma_{a}{}^{d}$ are the dispersion component and polar component of the asphalt, respectively (mJ/m^2^).

#### 2.3.4. Contact Angle Test of Asphalt Samples

**Figure 2:**
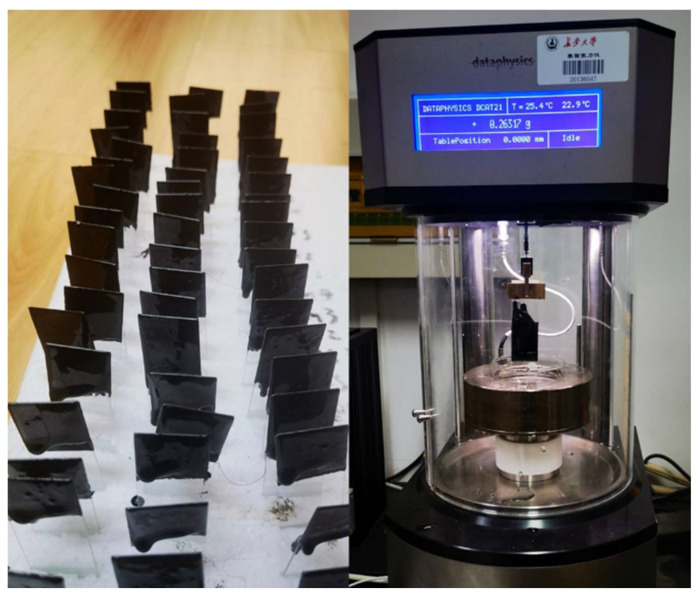
Dynamic surface tensiometer.

The surface free energy of asphalts with different rubber powder contents was tested by using a German DataPhysics dynamic surface tensiometer, as shown in Figure 2. A glass slide with a flat asphalt film was formed by the Wilhelmy hanging plate method, and the contact angle of the asphalt was measured [39]. The surface free energy of the asphalt was quantified using two liquids with known surface energies that are insoluble in asphalt and do not chemically react with asphalt. The test was conducted at a temperature of 25 °C, and each set of experiments was run in parallel with three times to rule out accidental errors.

#### 2.3.5. Contact Angle Test of Aggregates

The contact angle of the aggregates was tested based on the static contact angle, and three probe solutions of distilled water, ethylene glycol, and glycerol were also used. The surface free energy parameters of the aggregates were calculated according to the abovementioned surface energy theory. Before the aggregate contact angle test, 200-mesh, 400-mesh, and 1000-mesh sandpapers were used to preliminarily grind one side of the aggregate slices to avoid contact angle lag caused by the rough surface of the aggregates. In this test, a JC000D1 contact angle tester was used for testing, and the experimental temperature was 25 °C. After the contact angle test and calculation, the SFE parameters of the three aggregates were obtained.

#### 2.3.6. Atomic Force Microscopy (AFM)

**Figure 3:**
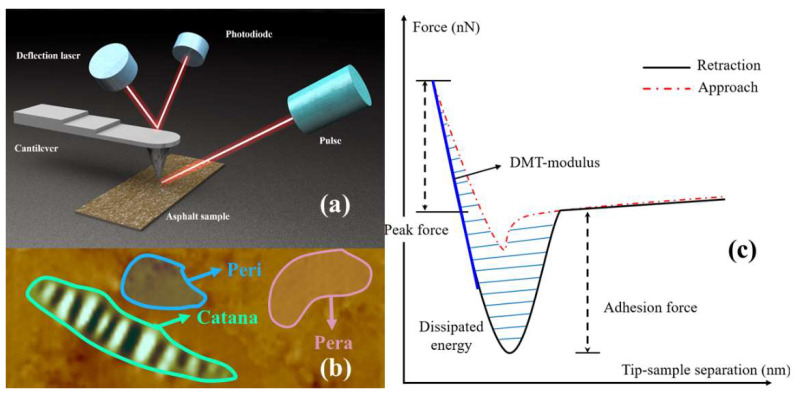
Operational principles of AFM: (**a**) operational principle of AFM; (**b**) schematic diagram of the three phases; (**c**) schematic force curve.

Microscopic images and nanomechanical characterizations of the four asphalt surfaces were obtained using a Bruker Dimension Icon Atomic Force Microscope (AFM), as shown in Figure 3a. The selected probe cantilever was a TAP300-G with a thickness of 4 μm, a width of 30 μm, a length of 125 μm, a nominal spring constant of 40 N/m, and a nominal resonance frequency of 300 kHz. The probe was uncoated, and the probe tip was made of monolithic silicon. The scanning frequency of the probe was set to 1.0 Hz, and different kinds of asphalt surfaces were scanned in tapping mode to obtain 20 μm × 20 μm topographic images and force curves of the asphalt surfaces [47]. Typically, three primary microstructures developed on the asphalt surface at around room temperature after annealing of asphalt from its melting temperature. The wrinkled areas were named the catanaphase (bee structures), the islands around the wrinkled domains were called the periphase, and the paraphase was the smoother phase neighboring the periphase, as shown in Figure 3b. The AFM images were analyzed by using the software Nanoscope Analysis 1.9 to quantitatively calculate the roughness of the samples. The adhesion force was determined from the measured force curve, as shown in Figure 3c.

#### 2.3.7. Cohesive and Adhesive

**Figure 4:**
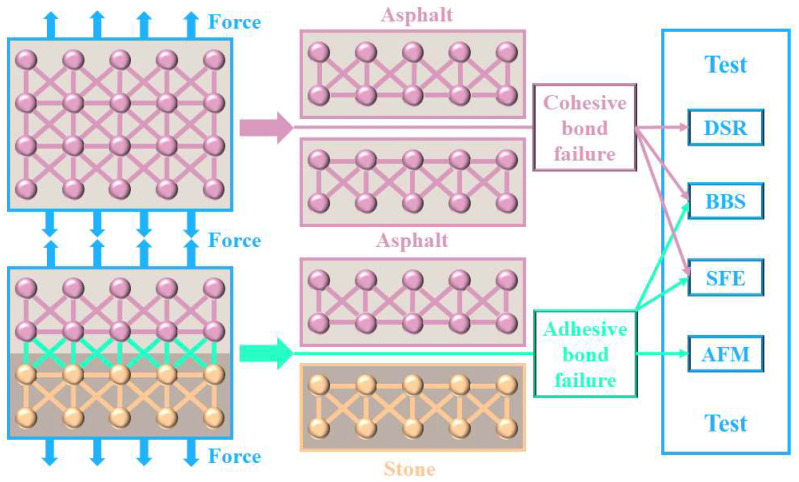
Cohesive and adhesive bond failure.

As shown in Figure 4, the cohesion energy or cohesive bond energy is defined as the value of the energy needed to create two new surfaces with unit areas [43]. The amount of energy required for deboning the binder–aggregate interface of the asphalt is called the adhesion energy (or adhesive bond energy) [43,44].

## 3. Results and Discussion

### 3.1. Analyses of Rheological Parameters

**Figure 5:**
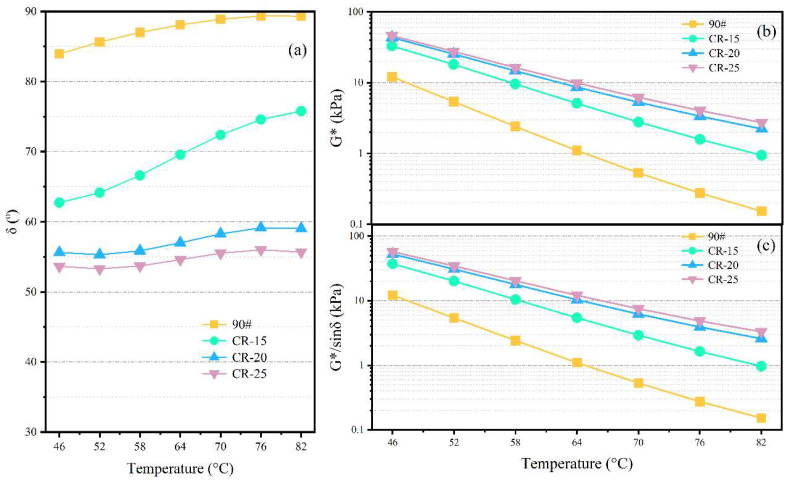
Results of the DSR test: (**a**) δ, (**b**) G*, and (**c**) G*/sin δ.

The rheological parameters of the four different binders under temperature sweep are shown in Figure 5. From Figure 5a, it can be seen that the phase angle of the base asphalt is relatively high, at 80–90° in the tested temperature range, while the phase angle of the rubberized asphalt is relatively low. The phase angle of CR-15 is within 60–80°, and it is greatly affected by temperature changes. The phase angle of CR-20 and CR-25 does not change much over the tested temperature range, within 50–60°. Unlike the base asphalt and CR-15, the phase angle of CR-20 and CR-25 decreases slightly within 76–82 °C. In general, the crumb rubber powder can reduce the phase angle of the binder.

Figure 5b shows the variation in the complex shear modulus for the different binders. It can be intuitively found from the figure that the complex shear modulus of the rubberized asphalt is several times that of the base asphalt at the same test temperature, and the gap is further expanded at high temperatures. Similar to the change in the phase angle, the complex shear moduli of CR-20 and CR-25 are similar, which indicates that 20% CR powder content can achieve a relatively stable CRMA system. The addition of CR powder can improve the complex shear modulus.

Figure 5c shows the variation in the rutting factor of different binders. The rutting factor can reflect the ability of the asphalt binder to resist permanent deformation at high temperatures. The variation in the rutting factor of the four binders is essentially the same as the variation trend of the complex shear modulus. In general, the rubberized asphalt improves the high-temperature rheological properties of the binder.

### 3.2. Pull-Off Tensile Strength (POTS) Analyses

**Figure 6:**
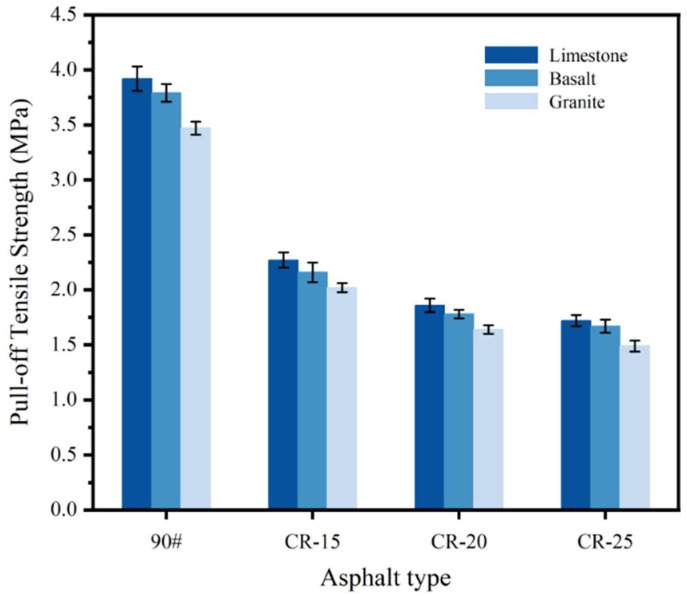
BBS test results of different rubberized asphalts.

The results of the POTS between the different rubberized asphalts and limestone, basalt, and granite are shown in Figure 6. It can be clearly seen from the figure that the CR powder can have a negative impact on the adhesion between the asphalt binder and the aggregate. Comparing the adhesion between the three kinds of rubberized asphalt and limestone, it can be found that the POTS of CR-15, CR-20, and CR-25 decreases by 42.1%, 52.6%, and 56.1%, respectively, compared with the base asphalt.

The addition of more CR powder has a greater negative impact on the adhesion performance of the asphalt binder, and similar results can also be found in SBS-modified asphalt [25]. The reason for these results is that additives such as CR powder can have a negative impact on the homogeneity of the asphalt. The CR powder is not inherently sticky, so it does not improve adhesion in the rubberized asphalt–binder system during POTS test. In addition, the CR powder has certain volume characteristics that are blended in physical form in the rubberized asphalt. The CR powder occupies a certain contact area at the interface between the rubberized asphalt and the aggregate, and the adhesion force brought by this part of the contact area is lower than the adhesion force between the asphalt and the aggregate, so the adhesion performance between the rubberized asphalt and the aggregate deteriorates [23].

There are also obvious differences in the adhesion between rubberized asphalt and different aggregates. It can be seen from the Figure 6 that the adhesion between rubberized asphalt and limestone is the best, followed by basalt, while the adhesion with granite is relatively poor. Taking CR-20 as an example, its POTS with granite is 1.64 MPa, and its POTS with basalt is 8.5% higher than that with granite, reaching 1.78 MPa, while its POTS with limestone is 13.4% higher than that with granite, reaching 1.86 MPa. The above results are caused by the differences in the properties of the different lithological aggregates, including the differences in the surface texture and composition of the aggregates [16].

### 3.3. Surface Free Energy (SFE) Analyses

**Figure 7:**
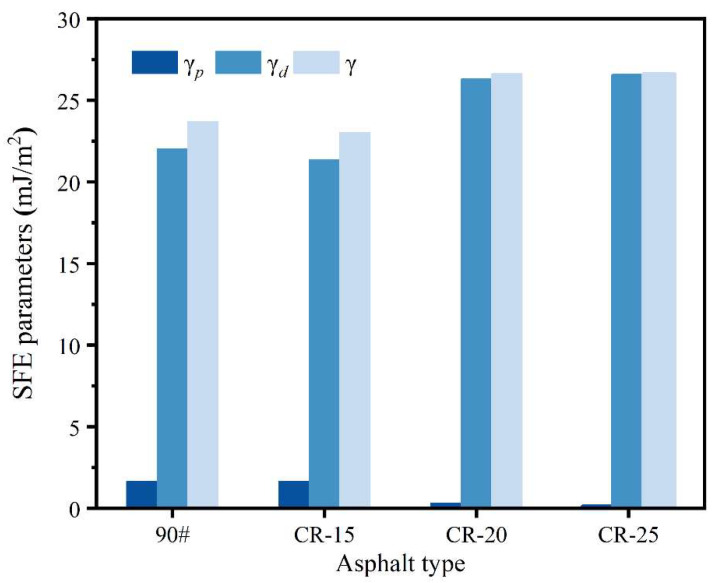
SFE parameters of different rubberized asphalts.

Figure 7 shows the calculation results of the SFE parameters of the four different asphalts. The polar components of the four asphalts are all much smaller than the dispersion components. Overall, the polar component decreases with the increase in dosage. The dispersive component and total SFE of the asphalts with the higher crumb rubber powder contents are higher. The total surface energy of the four asphalts is between 23 mJ/m^2^ and 27 mJ/m^2^. Surface energy theory states that the surface energy of a substance in a stable state is low. As shown in Equation (2), asphalt with a higher surface energy has higher cohesive energy. Based on the SFE parameters of the base asphalt and the three kinds of rubberized asphalt, 90# ≈ CR-15 < CR-20 ≈ CR-25.

The polar components of these four asphalts are 1.66, 1.667, 0.321, and 0.0968, respectively, and the change rule is a decreasing trend. Some scholars believe that the rubberized asphalt has a negative impact on the smooth glass slide, and samples with rough surfaces can easily lead to inaccurate test results [25]. In this work, the dynamic contact angle test method was used to continuously test the asphalt slides to avoid the uncertainty of a single static contact angle. With the increase in rubber powder content, the polar component decreases, the dispersion component increases, and the cohesive energy increases. Therefore, it is recommended to use the dynamic contact angle to test the SFE parameters of rubberized asphalt.

**Figure 8:**
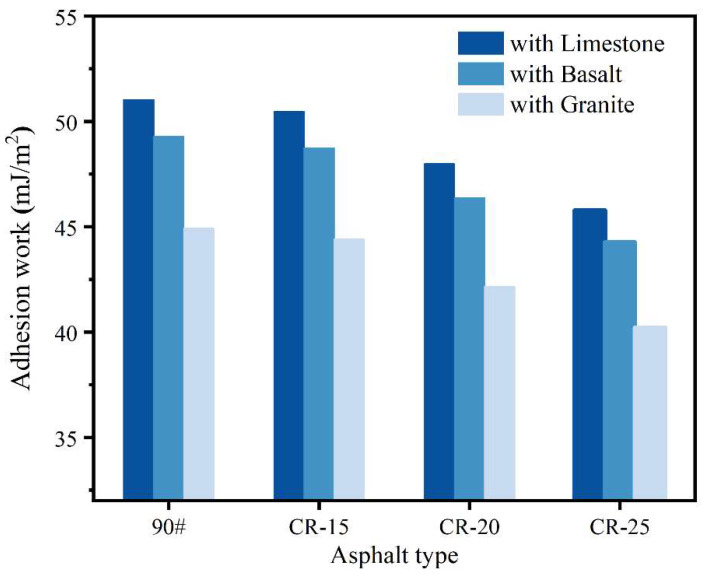
Work of adhesion of different rubberized asphalts.

From the SFE parameters of three kinds of aggregates, as shown in Table 4, it can be seen that the surface energy of limestone is greater than that of basalt, while the surface energy of granite is the lowest. The maximum polar component of limestone is 21.37 mJ/m^2^, and the minimum polar component of granite is 16.91 mJ/m^2^. Figure 8 shows the calculation results of the work of adhesion between the different asphalts and the three aggregates. The four kinds of asphalt have the strongest adhesion to limestone, followed by basalt, and the worst adhesion to granite. Taking the base asphalt as an example, the work of adhesion of base asphalt to limestone, basalt, and granite is 51.031 mJ/m^2^, 49.291 mJ/m^2^, and 44.917 mJ/m^2^, respectively. As an acidic substance, the adhesion of asphalt to the aggregate is greatly affected by the acidity and alkalinity of the aggregate. The adhesion between alkaline aggregates and asphalt is better than that of acidic aggregates [48].

In addition, from the perspective of asphalt, the addition of CR powder can reduce the work of adhesion between the rubberized asphalt and the aggregate, thereby weakening the adhesion performance. Taking the work of adhesion between the different asphalts and basalt as an example, the work of adhesion between the base asphalt and basalt was 49.29 mJ/m^2^, while the work of adhesion between CR-15, CR-20, and CR-25 and basalt was 48.75 mJ/m^2^, 46.38 mJ/m^2^, and 44.25 mJ/m^2^, respectively. The adhesion of CR-15, CR-20, and CR-25 to basalt was 1.10%, 5.91%, and 10.22% lower than that of base asphalt, respectively. The reason for this is also because the crumb rubber powder, which does not have adhesive properties, occupies a certain area of the adhesive interface between the asphalt and the aggregate.

### 3.4. Micromorphological Analyses

**Figure 9:**
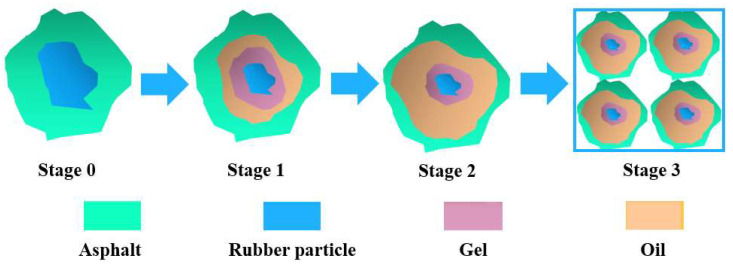
The asphalt–rubber interaction stages.

The modification with crumb rubber particles absorbed the light components of the asphalt and swelled, forming a uniform interconnection network in the asphalt system [49]. Rubber powder modification has a mainly physical effect [50]. The asphalt–rubber interaction stages can be divided into three steps, as shown in Figure 9 [51,52].

Stage 0—initial configuration: Rubber particles are immersed in the fluid asphalt. 

Stage 1—swelling phase: Rubber particles start swelling by absorbing the light fractions of bitumen and form a gel layer adjacent to the bitumen–rubber interface. 

Stage 2—post-swelling and beginning of degradation: The swelling of the rubber particles continues. Meanwhile, chemical degradation takes place through the breakup of the crosslinked network and polymer chains. Swollen rubber particles are split into smaller ones due to the destruction of the network structure. 

Stage 3—degradation and complete dissolution: The degradation of the rubber particles continues progressing until they are completely dissolved into the bitumen matrix, which produces a homogeneous binder.

Regarding the microstructure of rubberized asphalt, some scholars believe that the dispersion of CR powder after absorbing the light components hinders the aggregation of asphaltenes, resulting in a reduction in the catanaphase and difficulty in identification [53]. However, there is evidence that an increase in the oil content can actually reduce the catanaphase [54]. Some studies suggest that asphaltenes do not play a decisive role in the formation of the catanaphase [28,55]. Thus, the reduction in light oil adsorption is related to the relevance of the reduced catanaphase is questionable. The investigation of the effect of crumb rubber on the microstructure using atomic force microscopy–infrared spectroscopy (AFM-IR) indicated that the main chemical change takes place in the paraphase [27], but that chemical change is not the main mechanism of rubber modification. Therefore, the nanomorphological changes in crumb-rubber-modified asphalt still tend to be physical changes caused by the unbalanced stress between phases.

**Figure 10:**
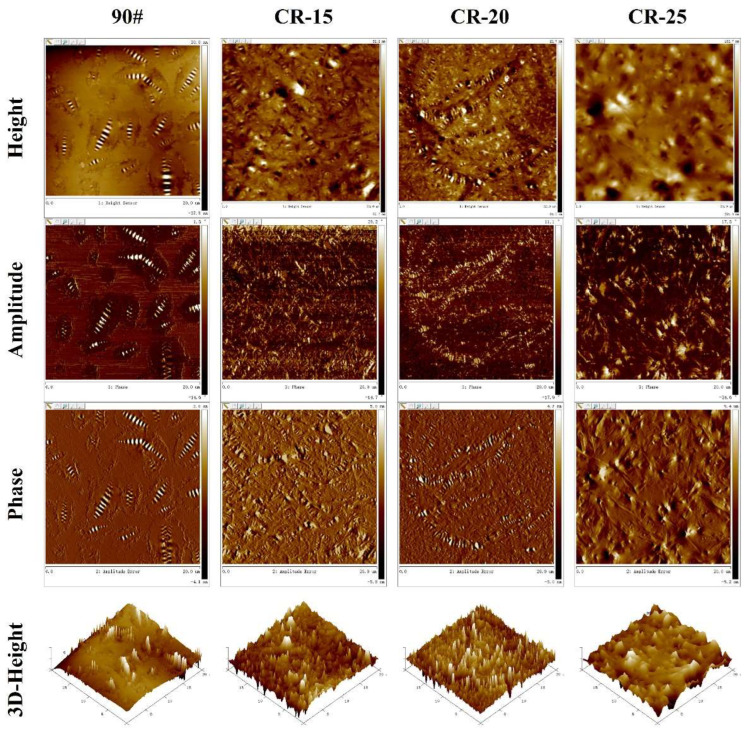
AFM diagrams of different rubberized asphalts.

Figure 10 shows the AFM images of different crumb rubber powder dosages. The catanaphase has obvious characteristic changes. The CR powder can be clearly seen in the three-dimensional image with the CR powder dosage of 25%. The change in the apparent structure may be more due to the change in the interfacial tension caused by the floating and agglomeration of the micro-rubber powder [56]. When the dosage is 15% and 20%, the catanaphase is broken; the details can be seen in Figure 10.

The common roughness indices are $S_{a}$, $S_{q}$, and $S_{Z}$, which are shown in Table 5 [57].

**Figure 11:**
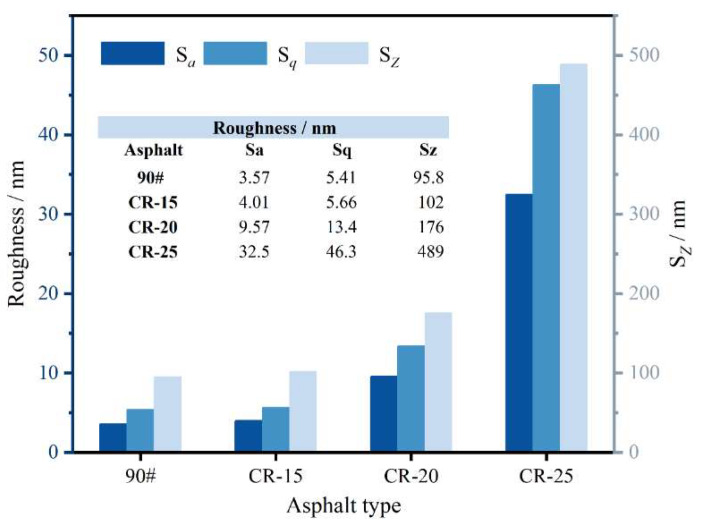
Roughness at different crumb rubber dosages.

As shown in Figure 11, it can be seen that the three indicators are consistent for the roughness changes with different dosages of CR powder. The smaller the roughness value, the smaller the difference between phases and the more stable the microstructural properties [58]. As shown in Figure 11, the roughness value is the highest and the microscopic morphology is the most unstable when the rubber powder content is 25%. The roughness value increases continuously with the increase in the rubber powder content. This shows that the content of rubber powder particles will continuously destroy the apparent morphology of the asphalt and reduce the stability of its microscopic properties. The roughness values did not change significantly at low rubber powder contents.

**Figure 12:**
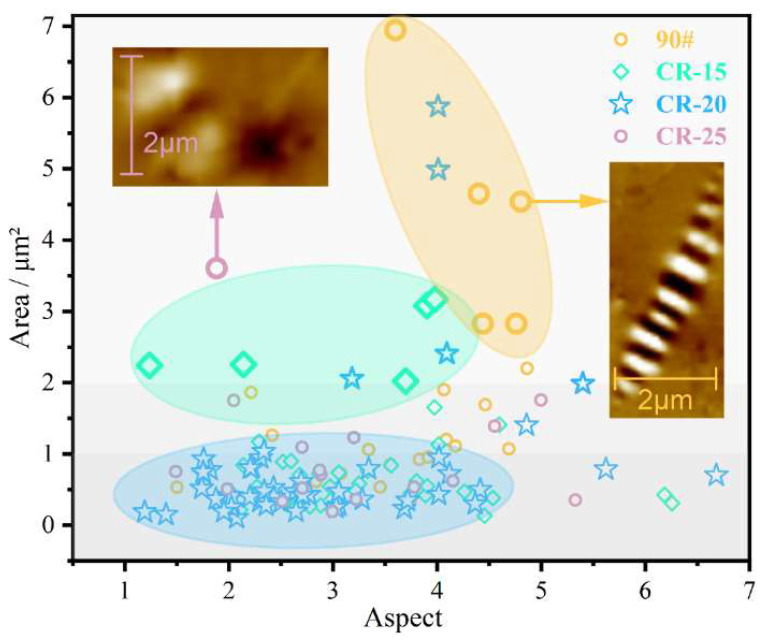
Morphological transformation of the catanaphase.

The schematic diagram of the scatter of morphological changes in the catanaphase is shown in Figure 12. The area is the area of the catanaphase, and the aspect is the ratio of the major and minor axes of the catanaphase. With the increase in the amount of CR powder, the slender catanaphase of the base asphalt becomes more dispersed when the amount of CR powder is 15%, and the length of the catanaphase also decreases. When the dosage is 20%, the original large catanaphase is broken into several smaller sections of catanaphase by the crumb rubber powder. When the dosage reaches 25%, the catanaphase effectively does not exist, and even if the catanaphase exists, it is blurred and counted in order to have a certain contrast, and it can actually be considered to be non-existent.

**Figure 13:**
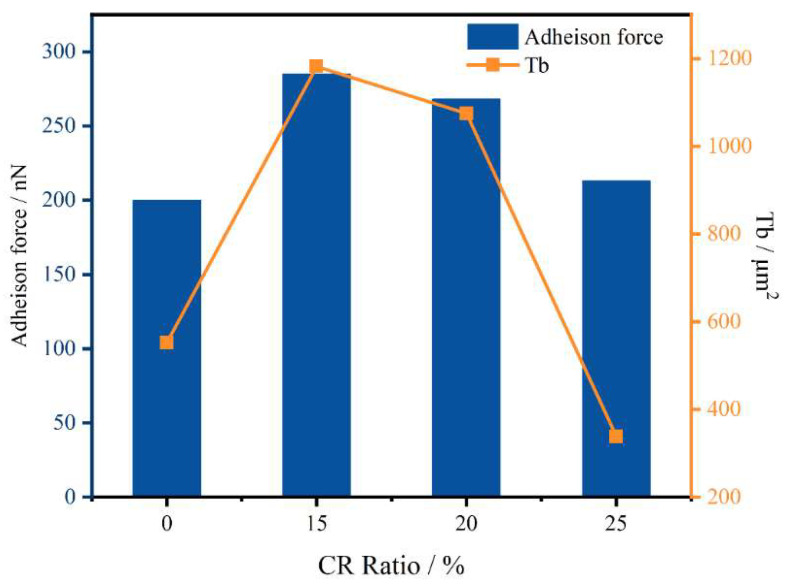
Adhesion force and catanaphase shape parameters.

As shown in Figure 13, the adhesive force shows a trend of first increasing and then decreasing with the increase in the CR powder content. Because the probe itself will be affected by van der Waals forces, the rubber forms a gel structure to improve the cohesion by absorbing the light components. The cohesion of the rubber-modified asphalt interferes with the test results, such that the adhesion of the rubber-modified asphalt is greater than that of the original asphalt. However, the comparison rule of rubber-modified asphalt is consistent with the previous test, indicating that atomic force microscopy is more suitable for the comparison of two-phase systems. When the dosage is higher, the CR microparticles can aggregate on the surface of the asphalt, destroying the surface tension. The stability of microscopic properties decreases, leading to a decrease in adhesion. In this process, the catanaphase is squeezed and broken by the continuously aggregated microgel powder particles until it completely disappears. The change trend of the catanaphase change coefficient Tb (as shown in Equation (8)) is consistent with the change in the adhesive force, indicating that the microstructure is strongly related to the performance, and the change in the catanaphase can be used to evaluate the modification effect of the CR powder.

(8) $$T_{b}=n\times {\bar{A_{s}}}^{3}$$

where *n* is the number of small catanaphase, and $\bar{A_{s}}$ is the average aspect of the catanaphase.

## 4. Conclusions

In this work, the adhesion characteristics of the original asphalt and three kinds of rubberized asphalt were studied. The rheological properties of the different asphalts were characterized by DSR, while the adhesion characteristics of the rubberized asphalts were analyzed from three perspectives by BBS tests, contact angle tests, and AFM. The following conclusions can be drawn:

1. The incorporation of CR can improve the complex shear modulus and reduce the phase angle of the asphalt. CR can significantly improve the stiffness, modulus, and cohesive energy of asphalt, thereby improving the high-temperature elastic properties of asphalt, which is also an important prerequisite for its wide application.
2. According to the BBS test results, CR has a negative effect on the pull-off tensile strength of asphalt and aggregates. CR itself does not have adhesive performance and can occupy part of the contact area at the interface between the asphalt and the aggregate, resulting in a deterioration in the adhesive properties of the rubberized asphalt.
3. CR can improve the SFE parameters of asphalt, and the changes in the total surface energy and dispersion components are significantly affected by the changes in CR content. The work of adhesion between asphalt and limestone is the highest, followed by basalt and, finally, granite, due to the differences in chemical composition between the different aggregates.
4. The large catanaphase of asphalt with higher CR contents is continuously broken with the increase in dosage. The roughness value increases with the increase in the dosage. CR-25 had the highest roughness value and the worst microscopic properties. The roughness values did not change significantly at low rubber powder contents.
5. The adhesion deteriorates with the increase in CR content. AFM is more suitable for the comparison of two-phase systems. The change factor of the catanaphase is consistent with the change trend of the adhesion force, and the microscopic morphology has a strong correlation with the change in the adhesion performance.

## 5. Further Study

In this work, a variety of test methods were used to evaluate the polymerization energy and adhesion energy of modified asphalt with different rubber powder contents, and the relationships between different test indices and cohesive energy and adhesion energy were explored. However, this work did not consider the effect of aggregate texture on adhesion performance, and it also did not explore the role of moisture. The evaluation of the adhesion properties of rubber powder still needs further verification.

## Figures and Tables

**Table 1:** Properties of the original asphalt.

Properties	Test Results	Test Methods
Penetration (25 °C, 100 g, 5 s; 0.1 mm)	92	T0604
Ductility (15 °C, 5 cm/min; cm)	>100	T0605
Softening point (°C)	46.5	T0606
Density (g/cm^3^)	1.023	T0603
Solubility (%)	99.71	T0607
Flash point (°C)	295	T0611

**Table 2:** Surface free energy parameters of the test liquids.

Type	γ*_d_* (mJ/m^2^)	γ*_p_* (mJ/m^2^)	γ (mJ/m^2^)
Distilled water	21.8	51.0	72.8
Ethylene glycol	29.3	19.0	48.3
Glycerol	34.0	30.0	64.0

**Table 3:** Chemical components of the aggregates.

Type	SiO_2_	Al_2_O_3_	Fe_2_O_3_	CaO	MgO	TiO_2_	Na_2_O	K_2_O	P_2_O_5_	MnO	Ignition Loss
Limestone	19.31	9.5	13.2	24.17	4.34	1.96	1.07	1.28	0.94	0.29	23.62
Basalt	47.32	15.0	16.36	8.21	3.76	1.44	2.13	0.44	0.21	0.17	1.94
Granite	72.05	12.81	2.13	0.75	0.11	0.07	2.81	4.63	0.04	0.02	2.88

**Table 4:** Surface free energy parameters of the three aggregates.

Type	γ*_d_* (mJ/m^2^)	γ*_p_* (mJ/m^2^)	γ (mJ/m^2^)
Limestone	17.43	21.41	38.64
Basalt	16.22	19.71	35.93
Granite	13.28	16.71	30.17

**Table 5:** Three-dimensional (3D) roughness parameters used in this study.

Parameter	Describe	Formula
$S_{a}$	Roughness average	$S_{a}=\frac{1}{A}\iint_{A}\left|Z(x,y)\right|dxdy$
$S_{q}$	Root-mean-square roughness	$S_{a}=\sqrt{\frac{1}{A}\iint_{A}\left|Z(x,y)\right|dxdy}$
$S_{Z}$	Maximum height of the roughness	$S_{Z}=S_{p}-S_{V}$
$S_{p}$	Maximum roughness peak height	$Z_{\max}$
$S_{V}$	Maximum roughness valley depth	$Z_{\min}$

## Data Availability

All data are contained within the article.
